# Adaptive traffic signal control using deep reinforcement learning: Toward smarter and safer urban mobility

**DOI:** 10.1371/journal.pone.0339207

**Published:** 2026-03-30

**Authors:** Fayez Alanazi, Ammar Armghan, Muhammad Tanveer, Amr Yousef

**Affiliations:** 1 Civil Engineering Department, College of Engineering, Jouf University, Sakaka, Saudi Arabia; 2 Department of Electrical Engineering, College of Engineering, Jouf University, Sakaka, Saudi Arabia; 3 School of Systems and Technology (SST), University of Management and Technology, Lahore, Pakistan; 4 Electrical Engineering Department, College of Engineering, University of Business and Technology, Jeddah, Saudi Arabia; 5 Engineering Mathematics Department, Faculty of Engineering, Alexandria University, Alexandria, Egypt; University of Shanghai for Science and Technology, CHINA

## Abstract

In today’s rapidly evolving Intelligent Transportation Systems (ITS), traditional systems for controlling traffic signals are often inadequate in optimizing real-time traffic flow due to their dependency on preset schedules and lack of adaptability to dynamically changing traffic signal phases. These systems cannot analyze dynamic signal timing changes, especially at multiple intersections, resulting in inefficient vehicle flow, longer queues, and higher levels of congestion. Thus, the need arises to develop intelligent systems capable of optimizing traffic flow in real time, reducing delays, and addressing the growing challenges of intelligent transportation systems. To address these requirements, a novel deep reinforcement learning framework that combines the Twin Delayed Deep Deterministic Policy Gradient (TD3) with prioritization-based Intelligent Traffic Control (P-ITC) is proposed for real-time traffic signal optimization using stability techniques. The module focuses on TD3’s stability-enhancing techniques, including clipped Q-learning, delayed and targeted policy updates, and smoothing. The system ensures robust signal timing decisions across intersection networks. PER prioritizes critical traffic signal experiences, ensuring the system learns from key events that influence real-time traffic flow. The proposed TD3P-ITC framework achieves maximum reductions in queue length (up to 22 at transport hub intersections and 25 at highways) and a 17.9 percent decrease (compared to baseline approaches) in simulated accident rates.

## 1. Introduction

Intelligent Transportation Systems (ITS) are becoming increasingly vital in addressing the growing challenges of vehicle congestion in urban environments [1]. Traffic signal control plays a crucial role in enhancing vehicle flow and mitigating queues at intersections [2]. Traditional frameworks fail to adapt dynamically to signal timing adjustments, thus requiring an intelligent approach to optimize [3]. In recent years, adaptive optimal control of traffic signals has attracted considerable attention in ITS, with RL emerging as a means to enhance traffic management efficiency and intelligence [4]. A key goal of ITS is to reduce queue length and throughput of traffic flow, with traffic signal control systems playing a vital role in optimizing traffic management at intersection points [5]. Recent advancements in DRL have significant potential to address the challenges of adaptive traffic signal control by learning optimal control policies from real-time traffic data [6]. With the growing urban traffic congestion, DRL dynamically optimizes traffic timings, thereby enhancing the efficiency of ITS [7]. For traffic signal control action space handles communication delays, thereby ensuring adaptability and scalability for real-time deployment in ITS [8]. A DRL-based real-time traffic analysis is employed, integrating real-time driving style attributes to optimize traffic flow and reduce computational requirements [9]. Building on this approach, the framework incorporates real-time passenger occupancy data in a transit signal priority, further enhancing its ability to minimize delay while optimizing vehicle flow efficiency [10].

RL-based traffic signal methods can efficiently learn from data but often require initial training with simulated data and subsequent fine-tuning with real-world inputs for optimal performance [11] by continuously updating Q-values by forecasting future traffic conditions, enabling the agent to adapt and optimize signal control strategies in real-time, ultimately enhancing intersection efficiency in ITS [12]. In DRL-based traffic signal control, the agent observes the traffic environment and makes decisions through either discrete or continuous action spaces to optimize signal control [13]. By integrating with policy updates in mixed-traffic environments, where both connected and non-connected vehicles coexist, it outperforms others by achieving the minimum average delay [14] with the focus on achieving training stability and faster convergence by guaranteeing the use of Double Deep Q-Networks (DDQN) and PER in the agent environment [15]. By incorporating expert guidance, DRL enhances the adaptability of traffic management in adaptive multiple-intersection environments [16]. The distributed agent-based DRL focuses on large-scale traffic signal control, leveraging the potential of multi-agent systems, and optimizes traffic flow across urban areas [17]. Due to the rise in urban populations and increasingly congested roadways, there is a demand for ITS that uses real-time and advanced algorithms to optimize traffic signal control, improve mobility, and mitigate the environmental impacts of congestion. The novelty of the TD3P-ITC framework lies in integrating DRL and PER to optimize traffic signal decisions. An integration of stability-enhanced techniques, such as target smoothing, ensures reliable decision-making. The key focus of this research includes:

- Designing a TD3P-ITC system optimizes signal timings across multiple intersections simultaneously, ensuring smooth traffic flow and reduced delays in urban networks.
- Applying PER helps focus learning on critical traffic scenarios, improve learning efficiency, and accelerate optimal decision-making in control.
- Integrating delayed policy updates and target smoothing reduces overestimation bias, ensuring stable and reliable signal control in dynamic traffic conditions.
- Balances key goals such as minimizing wait time, maximizing throughput, reducing congestion, and ensuring safety.

## 2. Recent works

Fereidooni et al. [18] utilize DRL algorithms, specifically DQN for SADRL and PPO for MADRL, as well as a real-time actuated control system (SMART) integrated with SUMO’s TraCI protocol. The dataset, sourced from the Snap4City platform, contains actual traffic data from Florence, Italy, under normal, medium, and heavy congestion conditions. With SMART obtaining the best tram/BRT prioritizing and the lowest Mean Travel Time (MTT), the results demonstrate that DRL-based algorithms perform better than Webster, SUMO, and MaMoTLO. The computing cost of real-time models like SMART is considerable, and there has been limited study of hybrid models that combine fixed-cycle efficiency with actuation adaptability. Another area where research is lacking is in scaling to larger urban networks.

Li et al. [19] presented a Federated PPO-based algorithm for intelligent traffic signal control spanning multiple domains, enabling secure, distributed joint training across many crossings. The dataset includes simulated traffic flow data from common crossings. Compared with the individual PPO, the results demonstrate a 27.34% decrease in vehicle waiting time and a 47.69% faster convergence rate. However, there is a lack of investigation into its practical application in fast-paced urban settings, as well as a corresponding lack of information about intersections. Wang et al. [20] improved junction modeling; this research proposes a multi-layered graph-masking-based learning approach for ATSC with many intersections. This algorithm combines upper and lower graph structures. Results demonstrate excellent scalability and generalizability, surpassing current approaches in traffic optimization and delay reduction. The dataset encompasses both real-world and synthetic urban road networks. Incorporating pedestrian data and improving graph layers, such as lane graphs, for more detailed modeling will be the focus of future effort, while handling intersections with more than four directions remains an unexplored area of research.

To improve both the length and phase selection of traffic signal control, Bouktif et al. [21] presented the MP-DQN framework, a parameterized DRL architecture. Results demonstrated a 33% improvement in travel time compared to traditional methods and a 7.5% improvement compared to GA and PSO approaches in the dataset, which was derived from simulations conducted in the SUMO environment. However, there are few studies on scaling the MP-DQN model for real-time deployment in large, multi-intersection networks, due to its increased computational complexity.

Zhang et al. [22] achieved a balance between efficiency, safety, and environmental effect. This study proposes a new method for real-time adaptive traffic signal regulation based on DRL. The methodology outperforms existing methods by combining the Dueling Double Deep Q Network (D3QN), resulting in a 16% reduction in vehicle congestion. The trade-off demonstrates the advantages of optimizing numerous targets, despite a little increase in waiting time (0.64%). The framework’s contributions to smarter, more sustainable traffic control solutions are particularly evident in high-traffic situations, where it delivers significant advantages. Cao et al. [23] employed SUMO for simulated urban traffic; this research presents an optimization strategy for traffic signals based on the Deep Q Network (DQN) algorithm. Compared with DQN and A2C, G-DQN achieves better results in terms of vehicle queue length and time, especially during peak hours. This is due to its enhanced traffic state representation and network convergence. Future research should improve deep reinforcement learning algorithms, such as A3C and TD3, to handle complex traffic situations and address multi-intersection scenarios, as this study only covers single-intersection models.

Cai & Wei [24] proposed an improved technique for controlling traffic signals based on DRL, incorporating a Dueling Double Deep Q-learning Network (D3DQN), a noise network, and priority experience replay (PER) to enhance the model and accelerate convergence. Significant improvements in queue length and waiting time, and faster convergence, were observed in results using a dataset comprising simulated traffic flow data from several crossings. However, there is a lack of studies on how to adapt the strategy for multi-agent collaborative control in real-world applications and on addressing the cold-start problem during early training. To address scalability and intersectional variability, Bao et al. [25] proposed Federated Learning-based Reinforcement Learning (FL-RL). The method incorporates local agent information into a global model. With real-world traffic data from Monaco included in the dataset, we can observe significant improvements in traffic flow efficiency and a 64.48% mitigation in total waiting time. When it comes to improving model performance and adaptability in varied urban traffic situations, there is a lack of research on sophisticated aggregation methods and meta-learning techniques.

An adaptive traffic control system, the M2SAC framework, was introduced by Zhang et al. [26], which uses a multi-agent-based masking of the soft actor-critic model to optimize signal light timing. Compared with baseline approaches and more conventional models, such as Webster’s formula, the dataset of real-world traffic scenarios from Melbourne, Australia, demonstrated an improvement of 5.17%. Problems with the model’s scalability in larger, more complex metropolitan settings, as well as the computational challenges of deploying it in real time under dynamic traffic conditions, warrant further study. Yazdani et al. [27] expanded the reward function to account for road user interactions and optimized automobile and pedestrian traffic flow using a Deep RL technique with Double Deep Q-Networks (DDQN). Using actual traffic data from the SCATS system improves vehicle travel time by 9% and reduces total user delays by 5%, particularly in scenarios involving high pedestrian volumes Table 1.

**Table 1:** Summary of related works on traffic control features.

Factors	[18]	[19]	[20]	[21]	[22]	[23]	[24]	[25]	[26]	[27]
Algorithm	DQN (SADRL), PPO (MADRL)	Federated PPO	Multi-layer Graph Mask Q-Learning (MGMQ)	MP-DQN	D3QN	DQN	D3DQN	FL-RL	Multi-agent Masked DRL (M2SAC)	DDQN (SADRL)
Dataset	Snap4City (Florence, Italy)	Simulated traffic data	Real-world and synthetic data	SUMO simulations	Real-world data (Melbourne)	SUMO (single-intersection)	Simulated traffic data	Real-world data (Monaco)	Real-world data (Melbourne)	SCATS system (pedestrian-heavy)
Real-Time Control	✔	✔	✔	✔	✔	✔	✔	✔	✔	✔
Traffic Flow	✔	✔	✔	✔	✔	✔	✔	✔	✔	✔
Vehicle Waiting Time	✔	✔	✔	✔	✔	✔	✔	✔	✔	✔
Scalability	✘	✘	✔	✘	✔	✘	✘	✔	✔	✘
Real-Time Adaptation	✔	✔	✘	✔	✔	✔	✔	✔	✔	✔
Computational Cost	✔	✔	✘	✔	✘	✔	✔	✔	✘	✔
Environment	✘	✘	✘	✘	✔	✘	✘	✔	✔	✔
Learning Stability	✔	✔	✔	✔	✔	✔	✔	✔	✔	✔
Traffic Complexity	✔	✔	✔	✔	✔	✔	✘	✔	✔	✔
Generalizability	✔	✔	✔	✘	✔	✔	✔	✔	✔	✔
Research Gaps	Scalability in large networks.- high computational cost in real-time models.	Limited real-world testing.- scalability for complex networks is not addressed.	Limited to junction models.	Real-time scaling challenges	Slight increase in waiting time.	Single-intersection focus.- limited generalization to multi-intersection setups.	Cold-start problem.- need for real-world implementation and scalability.	Lack of dynamic signal update	High computational cost with multi-agent systems.- real-time scalability in large urban settings.	Limited to single intersections.- lack of adaptability to more complex systems.

Zhao et al. [28] proposed a signal control optimization model for overflow prevention during nonrecurrent congestion, when the traffic volume and the bottleneck capacity are unknown, as in the case of traffic accidents. The approach anticipates available space in the remaining lanes of exit and arrival-departure curves based on partial vehicle connectivity information. It recalculates signal timing using a model predictive control system. Case studies show that overflow prevention is highly effective when connected vehicle penetration rates exceed 10 percent. The average delay reduction is 48.56 percent and 24.49 percent relative to adaptive signal control approaches that did not consider exit-lane conditions and those that did not consider a predictive model, respectively. A collaborative model that determines the quantity, width, type, distribution, and signal settings of the separated junctions to maximize capacity was introduced by Shi et al. [29] as a lane layout design and signal optimization model. Using modified saturation flow models, the method establishes relationships among three lane types: conventional-width lanes (CWL), special-width approach lanes (SWAL), and dedicated passenger-car lanes (DPCL). An optimization framework for mixed-integer quadratic programming is developed and tested, with road width as the primary input, using a branch-and-bound technique. With more adaptability and robustness for traffic groups of varying widths and structures, the numerical evidence shows that dedicated passenger car lanes and special-width lanes improve intersection capacity by an average of 12.51%.

K. Jalil et al. [30] provide a comprehensive review of collision avoidance (CA) devices in internet-connected vehicles (ICVs), focusing on sensor-based perception, communication technologies, and data-driven AI to enable real-time optimization. The paper also critically examines various CA techniques and their effectiveness in identifying and preventing static and dynamic barriers, as well as in preventing contact with other road users. Focusing on the value of integrated control systems, the review underlines the benefits of these technologies in enhancing vehicle performance, improving network-wide traffic efficiency, and preventing accidents, based on recent research and peer-reviewed materials. Current research on traffic signal control systems using DRL has several major limitations: they fail to achieve improved performance in real-time traffic networks, particularly at multiple intersections and with complex traffic signal phases. Numerous studies, such as Fereidooni et al. [18] and Li et al. [19], have demonstrated the efficacy of DRL models; however, they have not comprehensively addressed scalability and real-time implementation. There is also a significant lack of research on hybrid models that combine fixed-cycle economy with adaptive actuation. The suggested model addresses these problems by combining scalable DRL algorithms that enable real-time adaptation and by making it more generalizable across complex metropolitan networks. The proposed TD3P-ITC model addresses significant deficiencies identified in current research, including inefficient traffic flow, lengthy vehicle queues, varying weather conditions across extensive networks, elevated computational costs in real-time systems, and insufficient generalizability across multi-intersection configurations. The model is designed to perform well across urban networks by utilizing hybrid DRL approaches and offering real-time flexibility. This improves traffic flow and reduces delays. The model can handle complex traffic situations and provide a more long-term solution for adaptive traffic signal control by leveraging advanced learning techniques.

## 3. The proposed methodology

### 3.1. Problem formulation

Consider a real-time urban traffic network consisting of N = 5 intersections, as residential, commercial, highway, mixed-use, and transport hubs labeled as {1,2,3,4,5}, as locations where each intersection i observes traffic conditions through 12 distinct features sampled at 1-minute intervals over 2000 observations. These features include normalized traffic volume $\overset{i}{\underset{t}{V}}$, inferred queue length $\overset{i}{\underset{t}{Q}}$, one-hot encoded signal phase (red, green, yellow) $\overset{i}{\underset{t}{\Phi }}$, and weather conditions $W_{t}$. The objective of this framework is to optimize traffic signal control decisions across a multi-intersection network. The goal is to minimize the average vehicle waiting time $T_{w}$, maximize throughput efficiency $P_{t}$ minimize congestion and ensure safety. The model aims to adapt in real time to dynamic traffic conditions, weather variations, and emergency vehicle prioritization, ensuring efficient traffic flow, reduced delays, and optimized safety at intersections. The objective function is given in Equation (1).


$$L(u)=\sum_{t=\text{1}}^{T}\left[-\alpha T_{t}-\beta Q_{t}+\gamma P_{t}\right]$$ (1)

Subject to:$\left\{ \begin{array}{c} @cT_{t+\text{1}}=T_{t}+\Delta T-\Delta T_{dep}\\ Q_{t+\text{1}}=Q_{t}+A_{t}-D_{t}\\ P_{t+\text{1}}=P_{t}+\Delta P_{signal}\\ G_{t}\leq G_{max},R_{t}\geq R_{min}\\ Q_{t}\leq Q_{max}\\ P_{t}\leq P_{max} \end{array}\right.$

The proposed TD3P-ITC addresses the problem by using DRL and applies PER to prioritize critical traffic events, ensuring effective learning from high-volume traffic scenarios. The safety constraint is achieved by penalizing accident-prone scenarios and by adapting signal phases to reduce risk.

**Fig 1:**
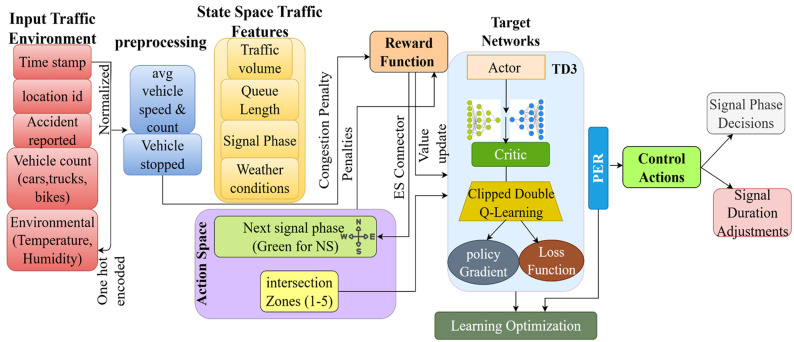
Overview of TD3P-ITC framework.

As shown in Fig 1, the TD3P-ITC framework controls traffic signals in intelligent transportation systems in real-time. It receives time-stamped vehicle counts, speeds, vehicle types, queue lengths, signal phases, and weather conditions. Normalized and pre-processed characteristics comprise the model’s decision-state space. A reward function is used to minimize congestion, maximize throughput, and ensure safety, with reward functions for accidents and traffic congestion. The action space includes signal phase decisions and their corresponding signal durations, which the TD3P algorithm optimizes based on traffic experiences. The model uses PER to prioritize essential traffic events for efficient learning and dynamically adjusts traffic lights in real time to enhance traffic flow across multiple intersections. The signal phase decisions for each intersection point are allotted and optimized through a policy update. The updated green-light duration in the NS and EW directions ensures balanced traffic flow and minimizes congestion.

### 3.2. Materials and methods

The dataset used for this research is a real-time urban traffic dataset from multiple intersections comprising 2000 observations and 12 distinct features [31]. The features are traffic metrics such as time-stamp, location ID (1–5) sampled at one-minute time intervals, traffic volume, vehicle counts for different types (cars, trucks, bikes), and environmental factors such as weather conditions (sunny, rainy, foggy, cloudy, and windy), temperature, and humidity. Also, the data source provides accident reports and the current traffic signal status (red, green, yellow). The Smart Traffic Management Dataset is an open-source dataset for intelligent transportation and traffic control, containing 2,000 time-stamped traffic observations with 12 variables per case. With aggregated observations of city-wide traffic sensors rather than data from a specific deployed city, the dataset provides a synthetic, open-source environment for research into traffic analytics and machine learning. There is no particular location associated with the dataset; thus, it is geographically generic.

The data is not in absolute calendar dates but is a time-indexed traffic record intended to capture representative changes in daily traffic, such as peak and off-peak periods. In this work, we first pre-process and transform raw traffic variables, including vehicle counts, average velocity, signal state, vehicle type distributions, and environmental variables, into a 12-dimensional state representation per intersection. From there, we derive traffic volume, queue length, and stop count from vehicle density and a low-speed threshold, inspect sensor data to obtain average velocity and speed variance directly, one-hot-encode signal phases, represent vehicle composition as proportional ratios, and numerically code and normalize weather, temperature, and humidity. The dataset does not contain any past accident records. However, to model the accident indicators needed for this work—such as traffic density, speed variation, signal conflicts, and bad weather—in the SUMO environment, which is used solely to create and assess safety-aware rewards. The dataset contains simulated safety incidents in the SUMO environment, activated by traffic congestion, velocity variance, signal crossing opportunities, and poor weather. Instead of simulating historical crash causality, these simulated accident indicators offer controlled, reproducible risk-minimization measurement through safety-conscious incentive shaping and performance evaluation.

#### 3.2.1. Pre-processing & traffic feature analysis.

**Fig 2:**
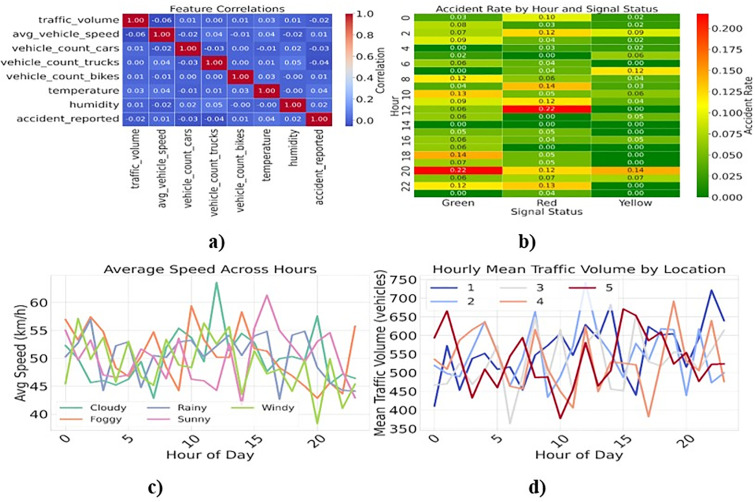
Traffic Environment Analysis a) Feature Correlation b) Accident Rate c) Average Speed Across Hours and d) Traffic Volume.

The raw traffic signal dataset undergoes several pre-processing steps to make it suitable for training the TD3P-ITC system. The time-based features, such as the hour $H_{t}$ and day $d_{t}$ are extracted from the time-stamp. For feature scaling, min-max normalization is applied to ensure uniformity across different metrics $\overset{i_{norm}}{\underset{t}{x}}=\frac{\overset{i}{\underset{t}{x}}-min\left(\overset{i}{\underset{t}{x}}\right)}{max\left(\overset{i}{\underset{t}{x}}\right)-min\left(\overset{i}{\underset{t}{x}}\right)}$ and adaptively applies z-score standardization for a zero mean and unit variance as $\overset{i_{std}}{\underset{t}{x}}=\frac{\overset{i}{\underset{t}{x}}-\mu_{i}}{\sigma_{i}}$. The feature extraction includes normalizing traffic volume $\overset{i_{norm}}{\underset{t}{V}}=\frac{\overset{i}{\underset{t}{V}}-\mu_{V_{i}}}{\sigma_{V_{i}}}$ with the queue length calculated based on traffic volume $\overset{i}{\underset{t}{V}}$ and average vehicle speed $\overset{i}{\underset{t}{Q}}=\frac{\overset{i}{\underset{t}{V}}}{\overset{i}{\underset{t}{Speed}}}$. Fig 2a-Fig 2d shows important parts of traffic management that utilize advanced analytics of traffic features. The correlation heatmap shows the relationships among key traffic parameters, including vehicle counts, average speed, and weather conditions. These features are important for constructing an optimum state space for signal management systems. The accident incidence heatmap examines accident rates by signal status and time. It demonstrates how changes in signal phase affect accident frequency. The combined subplots also provide a comprehensive view of traffic patterns. The average speed of vehicles in different weather conditions is shown, while the other chart displays the number of vehicles on the road at various times of day. These assessments work together to help plan real-time adaptive traffic signal control, thereby enhancing safety and improving traffic flow. The research emphasizes the necessity of dynamic signal adjustments.

#### 3.2.2. State space model.

The state space $s_{t}$ gives the current condition of the traffic environment at time t, including the key features that define the flow of traffic and environment at each intersection point. The state space is composed of the following elements using Equations (2) and (3):


$$s_{t}=\left[\overset{\text{1}}{\underset{t}{V}},\overset{\text{2}}{\underset{t}{V}},\ldots ,\overset{N}{\underset{t}{V}};\overset{\text{1}}{\underset{t}{Q}},\overset{\text{2}}{\underset{t}{Q}},\ldots .,\overset{N}{\underset{t}{Q}};\overset{\text{1}}{\underset{t}{\Phi }},\overset{\text{2}}{\underset{t}{\Phi }},\ldots ,\overset{N}{\underset{t}{\Phi }};W_{t}\right]$$ (2)

In contrast to the overall network state, Equation (2) describes the per-intersection state. Factors such as ambient variables, vehicle composition, average speed, predicted wait time, 12-dimensional feature vectors, and traffic volume are present at the intersection. All of these vectors, one for each of the N = 5 intersections in the network, add up to the global state. In this scenario, the state vector is 60 bytes, and the overall state dimension is 12N.


$$s_{t+\text{1}}=f\left(s_{t},u_{t}\right)\to statetransitionwithnewsignaltimings$$ (3)

Here, the normalized traffic volume at [i,t] is given as $\overset{i}{\underset{t}{V}}$ acts as a primary indicator of traffic congestion, and a high volume directly contributes to a higher negative reward, incentivizing the agent to clear traffic. The inferred queue length at [i,t] based on vehicle speed and count, it is given as $\overset{i}{\underset{t}{Q}}$, one-hot encoded signal at [i,t] is termed as $\overset{i}{\underset{t}{\Phi }}$ and $W_{t}$ represents the one-hot encoded weather that impacts traffic flow by reducing speed during rainy days, and fog increases the distance, scaled temperature, and scaled humidity. Table 2 summarizes the key hyperparameters used in the experiments to train the proposed TD3P-ITC framework, including network learning rate, exploration rate, prioritized replay, reward weighting, and convergence.

**Table 2:** State Space feature specification.

No.	Feature	Symbol	Source/ Derivation	Normalization	Rationale
1	Traffic volume	$v_{i,t}$	Raw vehicle counts per interval	Min–max normalization	Primary indicator of demand and congestion
2	Inferred queue length	$q_{i,t}$	Estimated from vehicle density and low-speed threshold	Min–max normalization	Directly reflects congestion severity
3	Average vehicle speed	${\bar{s}}_{i,t}$	Sensor-reported mean speed	Z-score normalization	Captures flow efficiency and stop–go behavior
4	Speed variance	$\overset{\text{2}}{\underset{s,i,t}{\sigma }}$	Computed from speed samples	Z-score normalization	Proxy for instability and accident risk
5	Stop event count	$\overset{\text{stop}}{\underset{i,t}{N}}$	Vehicles with speed ≈ 0	Min–max normalization	Penalizes excessive stopping
6	Signal phase (NS)	$\overset{NS}{\underset{i,t}{\varphi }}$	Signal controller log	One-hot encoding	Phase awareness without phase control
7	Signal phase (EW)	$\overset{EW}{\underset{i,t}{\varphi }}$	Signal controller log	One-hot encoding	Avoids unsafe phase ambiguity
8	Vehicle composition ratio	$\overset{\text{heavy}}{\underset{i,t}{r}}$	Truck/bus count by total vehicles	Min–max normalization	Accounts for heterogeneous vehicle behavior
9	Weather condition	$w_{i,t}$	Categorical weather label	One-hot encoding	Affects speed, safety, and capacity
10	Temperature	$T_{i,t}$	Environmental sensor	Z-score normalization	Secondary factor affecting traffic flow
11	Humidity	$H_{i,t}$	Environmental sensor	Z-score normalization	Correlated with weather-induced risk
12	Intersection identifier	${\text{ID}}_{i}$	Static intersection label	Integer embedding	Enables multi-intersection differentiation

The chosen properties ensure that control relevance, observability, and learning stability are all equal in the process. Optimizing a signal timing requires fundamental congestion indicators such as speed, queue length, and traffic volume. The sensitivity to stop-go vibrations and the safety risk can be represented by stop events and speed variance, eliminating the need to model actual collisions. Signal-phase encoding maintains deterministic phase control while also providing context-aware phase control. To estimate the effects of external disturbances on capacity and accident probability, we use environmental factors and vehicle composition to capture the heterogeneous discharge behavior. Pedestrian movement, turning ratios, and lane movements are not part of this model because they are either not included in the provided dataset, are highly intersection-specific, or would significantly increase the state’s dimensionality without being uniformly observable. They can employ their state representation in continuous-control reinforcement learning thanks to their exclusion techniques, which make it compact, scalable, and sensor-realistic.

#### 3.2.3. Action vector representation.

Action function $a_{t}$ represents the traffic signal control decisions based on current traffic signal phases, including red, green, and yellow, along with the duration of green lights across multiple intersections. It is represented as a vector containing the green and yellow durations for the North-South (NS) and East-West (EW) directions. The agent $a_{t}$ learns to choose the optimal signal phase or timing to minimize congestion and waiting times. The inclusion of signal phases and green light durations is formalized as in Equation (4). The time-varying action is determined by adjusting the duration of the continuous green phase at time t based on the current signal phase state.


$$a_{t}=\left[\varphi_{NS},\varphi_{EW},\Delta t_{NS},\Delta t_{EW}\right]anda_{t}=\text{arg}\underset{a_{t}}{\text{max}}Q\left({s_{t},a}_{t}\right)$$ (4)

Where signal phases $\varphi_{NS}$ and $\varphi_{EW}$ the signal phase for the North-South direction can be red, green, or yellow, and similarly for the East-West direction. $\varphi_{NS},\varphi_{EW}\in \left\{\text{Red},\text{ green},\text{ and yellow }\right\}$ as signal phase decision. The green durations are $\Delta t_{NS},\Delta t_{EW}\in \left\{\Delta t_{min},\Delta t_{max}\right\}$ are given for North-South and East-West directions, where $\Delta t_{min}$ indicates the minimum green light duration allowed, and $\Delta t_{max}$ as the maximum green light duration allowed to prevent excessive delays at the opposite intersection, the action space manages the length of the currently active green stage for North-South or East-West movement without optimizing discrete phase transitions. To ensure traffic safety, the signal controller enforces yellow phases and all-red clearing intervals automatically during all phase transitions. Each green-to-red transition begins with a yellow gap and complete red clearing before the opposing way is activated. The green time values $\Delta t_{min},\Delta t_{max}$are set based on traffic engineering and network saturation to prevent dangerous switching or severe hunger. All experiments limit the agent’s continuous action outputs to this permissible range, then apply the signal timing controller. Yellow and all-red phase safety restrictions are set outside the learning loop and not violated by policy. These two $\Delta t_{NS},\Delta t_{EW}$ represents the durations that mitigate congestion and maximize throughput while considering the traffic volume V at each intersection. Also, the $Q\left({s_{t},a}_{t}\right)$ represents the estimation of the expected penalty for taking $a_{t}$ in $s_{t}$. The agent selects the signal phase that yields the best trade-off between traffic flow optimization and safety. The proposed agent does not perform discrete phase selection, it selects only duration. External factors drive the establishment and imposition of signal phase sequencing to ensure compliance with safety and regulatory standards. Only activities that are constrained by the length of the running green stage are considered by the TD3 policy to be part of the continuous action space. There is no discretization or mapping of continuous actions to phase decisions, as the continuous outputs are fed directly into the timing control variables. This ensures policy stability and compatibility with continuous-control reinforcement learning.

However, the TD3 control strategy is unable to learn discrete phase selection; it can only learn temporal modifications of continuous values. To ensure safety and practicality, a predetermined operating sequence controls the signal phases, and the actor network generates continuous control signals that indicate whether the active green interval should be extended or shortened. To ensure full compatibility with continuous-action reinforcement learning, the phase terms in the action formulation refer to the current context of active control rather than the policy-optimized choice variables. The TD3P-ITC performs action updates to adjust the green timings dynamically if $Q_{L\text{1}}$ queue at lane 1 exceeds a threshold, increase $S_{L\text{1}}$ signal green time for lane 1. If $P_{L\text{1}}$ throughput for lane 2 is high, reduce $S_{L\text{2}}$ to avoid congestion in other lanes

#### 3.2.4. Reward function.

The reward function $r_{t}$ helps guide the DRL model in making decisions that optimize traffic flow, including minimizing queue lengths, maximizing throughput, and preventing accidents. This reward function ensures that the agent not only optimizes traffic flow but also prioritizes safety by discouraging accidents and reducing congestion at intersections in the traffic environment given in Equation (5).


$$r_{t}=\left\{ \begin{array}{c} @c-(\alpha \cdot \sum_{i=\text{1}}^{N}q_{i,t+\text{1}}+\beta \cdot \sum_{i=\text{1}}^{N}S_{i,t+\text{1}}+\gamma \cdot {\text{1}}_{accident,t+\text{1}})\\ Whereq_{i,t+\text{1}}=f_{queue}\left(V_{i,t+\text{1}},S_{i,t+\text{1}}\right)=\frac{qL_{i,t+\text{1}}}{V_{i,t+\text{1}}+\in }\cdot \left(\text{1}-\frac{S_{i,t+\text{1}}}{S_{max}}\right)\\ S_{i,t+\text{1}}=f_{stops}\left(V_{i,t+\text{1}},{speed}_{i,t+\text{1}}\right)=\frac{\overset{stop}{\underset{i,t+\text{1}}{N}}}{{speed}_{i,t+\text{1}}+\in }\\ {\text{1}}_{accident,t+\text{1}}=\left\{ \begin{array}{c} \text{1},ifP_{accident,t+\text{1}}\geq \theta_{acc}\\ \text{0},ifP_{accident,t+\text{1}}<\theta_{acc} \end{array}\right. \end{array}\right.$$ (5)

Where $q_{i,t+\text{1}}$ as the inferred queue length [i,t+1], and $S_{i,t+\text{1}}$ as inferred stops [i,t+1] based on vehicle speed and count, the accident reported at t+1 is given as ${\text{1}}_{accident,t+\text{1}}$. The queue length $L_{i,t+\text{1}}$ at intersection i at time t+1, the traffic volume $V_{i,t+\text{1}}$ at intersection i at time t+1, with $S_{max}$ as the maximum acceptable number of stops. In the stop penalty function,$\overset{stop}{\underset{i,t+\text{1}}{N}}$denotes the number of stopped vehicles at intersectioniat time t+1, and the term ${speed}_{i,t+\text{1}}$ represents the average vehicle speed. Here $P_{accident,t+\text{1}}$ represents the risk proxy, not the trained probabilistic classifier output. It is deterministically derived using normalized queue length, mean speed variation, traffic volume, weather condition indicators, and the current signal phase at time t. The accident risk threshold $\theta_{acc}$ determines the level of risk tolerance for safety penalties, which is a non-learnable hyperparameter. It uses visible traffic and environmental factors, such as normalized wait length, traffic density, speed fluctuations, weather conditions, and signal phase conflicts, to produce a closed-form risk measure. These traits are linearly concatenated and passed through a sigmoid function to generate a normalized value in [0,1] representing short-term accident risk, not a learning probability model.

The incentive weights (α (Queue penalty weight), β (Stop penalty weight), γ (safety/accident weight)) were determined through empirical sensitivity analysis rather than heuristic estimates. After factoring in congestion reduction, stop minimization, and safety penalties, a coarse grid search of permissible ranges was fine-grained to train stability and convergence rate with respect to policy strength. The chosen weights steadily improved performance without causing oscillatory signals or safety breaches. After identification, the weights were held constant across all intersections, traffic demand levels, and weather to ensure fair comparison and demonstrate the policy’s generalizability. Training and evaluation were not weighted per intersection or scenario-reward.

**Fig 3:**
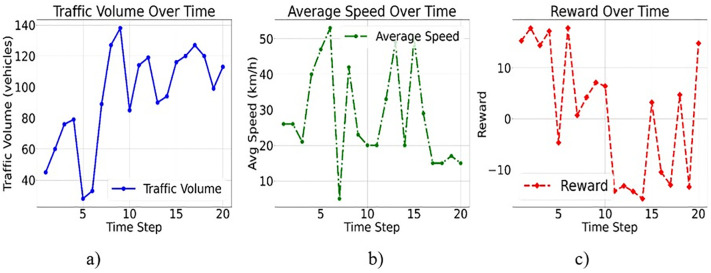
Traffic Control Analysis Over Time Across a) Traffic Volume, b) Average Speed, and c) Reward Function.

Fig 3a-3c reports that the accident acts as a primary reward signal and safety indicator for the PER mechanism. A value of 1 triggers a large negative reward, ensuring that the agent quickly learns to avoid accident-routine scenarios. The signal status from agents’ outputs determines the next signal phase, as transitioning from red to green indicates control over the environment. In this simulation, the agent adjusts the signal phases at a traffic intersection based on weather conditions, accident reports, and traffic volume. The three plots show how the agent’s choices affect traffic over time. For example, traffic volume varies with weather conditions and signal phases, peaking during rush hours, and accidents exacerbate the issue. Second, the average speed follows a similar trend, slowing down when there are accidents or adverse weather conditions, such as rain or fog. Finally, the reward function indicates how effectively the agent achieved these objectives. Higher payouts mean better traffic management and fewer accidents.

TD3 trains a deterministic policy in an off-policy manner, where agents learn to explore on-policy features in the learning environment, with dynamic updates driven by various learning signals. The TD3 framework uses double Q functions via mean-squared Bellman error minimization, which differs from other learning models in how it updates the dimension of each action function. Adding the noise to each dimension of the $a_{t}$ function validates the penalty rewarded for learning and ensures that the target actions are taken. The target actions follow stability techniques to provide a valid action range and corresponding reward update.

#### 3.2.5. Clipped double Q-learning.

The traffic signal control model is implemented by combining clipped double Q-learning and TD3 to optimize the agent’s decision-making, minimizing bias in Q-value estimates and stabilizing learning across traffic states during intersection analysis. TD3 utilizes two Q-functions, Q1 and Q2, and selects and evaluated at each decision point. The target Q-value for a transition is given as $\left(s_{t},a_{t},r_{t},s_{t+\text{1}},r_{t}\right)$ a sequential transition tuple, with the initial state $s_{t}$, the executed action $a_{t}$, intermediate reward $r_{t}$, γ as the discount factor and the subsequent state $s_{t+\text{1}}$ and $d_{t}$ as a binary indicator for episode termination. The Q-value target is computed as in Equation (6),


$$y_{t}=r_{t}+\gamma (\text{1}-d)\cdot min\left(Q_{\text{1}}\left(s^{\prime },a^{\prime }\right),Q_{\text{2}}\left(s^{\prime },a^{\prime }\right)\right)$$ (6)

Target policy ensures smoother policy updates and prevents overfitting to Q-values, which might lead to instability. With the gradual update of the target network, the action decisions are made by the actor, for instance, the green duration for signals, which allows for stability and avoids overreacting to transient traffic fluctuations, and $a^{\prime }\left(s^{\prime }\right)$ as the noisy target action. To prevent the policy from exploiting sharp, high peaks in the Q-function, it is expressed as in Equation (7),


$$a^{\prime }\left(s^{\prime }\right)=clip\left(\mu \theta_{target}\left(s^{\prime }\right)+clip(\in ,-c,c),a_{low},a_{high}\right),\;\in \sim N(\text{0},\sigma )$$ (7)

The function $a^{\prime }\left(s^{\prime }\right)$ smoothes the decision-making process, helping the agent avoid overreacting to transient traffic changes. Here N(0,σ) is a Gaussian noise and clip(∈,−c,c) is a clipping bound, where ∈ is a random noise that smoothes and helps average out Q-value errors over similar actions. The term $\mu \theta_{target}\left(s^{\prime }\right)$ is the target action policy based on traffic features like weather and crash type, evaluated at the state $s^{\prime }$. From the actor network for the state $s^{\prime }$ followed by $a_{low},a_{high}$ implies the lower and upper bounds for current signal phase durations and traffic risk policies, such as safety measures.

**Fig 4:**
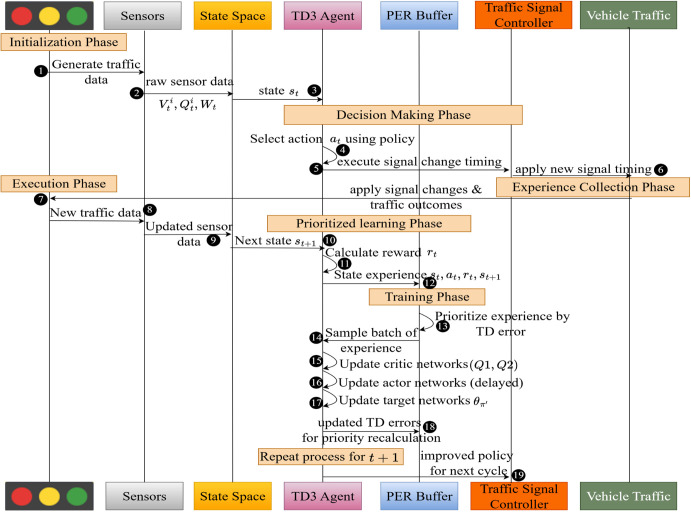
System interaction sequence flow of TD3P-ITC.

Fig 4 illustrates the complete process of the TD3P-ITC framework, which is utilized to manage traffic signals in a manner that adapts to changing conditions. The first step is to generate traffic data and a state space from sensor inputs, including vehicle counts, queue length, and wait time. The TD3 agent uses a policy to choose actions that modify the timing of signals and then implements those changes. The experience collection phase collects new states and rewards, then places them in the PER buffer. After that, the agent’s experience is prioritized based on the TD error, and the critic and actor networks are then updated during training. The framework continually improves its signal control policy, which enables it to work more effectively over time.

#### 3.2.6. Target network and delayed updates.

The target networks provide stability by slowly maintaining the rewards of both actor and critic networks that generate consistent learning target stages, which prevents the moving target problem in the earlier RL models using the soft update mechanism $\theta_{\pi^{\prime }}\leftarrow \tau \cdot \theta_{\pi }+(\text{1}-\tau )\cdot \theta_{\pi^{\prime }}$. Along with delayed policy updates, enhancing this stability involves updating the actor network every 𝑑 = 2 step while critics update continuously, allowing Q-value estimates to converge near their true values before policy modification. This plays an important role in traffic scenarios where premature signal changes can cascade through the network. The soft update rule for the target network is given by Equation (8).


$$\begin{array}{c} \theta_{\pi^{\prime }}\leftarrow \tau \cdot \theta_{\pi }+(\text{1}-\tau )\cdot \theta_{\pi^{\prime }}\\ \theta_{{Q\text{1}}^{\prime }}\leftarrow \tau \cdot \theta_{Q\text{1}}+(\text{1}-\tau )\cdot \theta_{{Q\text{1}}^{\prime }}\\ \theta_{{Q\text{2}}^{\prime }}\leftarrow \tau \cdot \theta_{Q\text{2}}+(\text{1}-\tau )\cdot \theta_{{Q\text{2}}^{\prime }} \end{array}\}$$ (8)

The policy network or parameters of the target policy update $\theta_{\pi^{\prime }}$ and the current policy network is given as $\theta_{\pi }$. The term $\theta_{{Q\text{1}}^{\prime }}$ and $\theta_{{Q\text{2}}^{\prime }}$ are the parameters of the target-function networks Q1 and Q2, with $\theta_{Q\text{1}}$ and $\theta_{Q\text{2}}$ are the parameters of the current Q-function networks, with τ as the update factor that controls the traffic. A small τ means the update is gradual, ensuring stable learning. The $\tilde{y}\left(r_{safe},s^{\prime },d\right)$ analyzes the minimum Q-value from two Q-functions given in Equation (9).


$$\tilde{y}\left(r_{safe,}S^{\prime },d\right)=r_{safe}+\gamma (\text{1}-d).\underset{i=\text{1},\text{2}}{\underbrace{min}}Q_{\alpha_{i,target}}(S^{\prime },a_{target}{\left(S^{\prime }\right)}^{\circ })$$ (9)

The term $r_{safe}$ is the safety reward for safe driving behavior, 𝛾 as the decay factor controlling the importance of future rewards, and 𝑑 as a flag indicating whether the traffic incident has concluded (1) or not (0). The parameter $Q_{\alpha_{i,target}}$ is the two Q-functions are used to evaluate traffic risks.

#### 3.2.7. Loss function.

For each Q function, calculate the predicted Q-values and the observed target value variations, to represent the traffic risk estimation using $\tilde{y}\left(r_{safe},s^{\prime },d\right)$ as target value, then square the differences to get the error terms for both Q-functions. For calculating the loss function, the squared errors over a B from the PER is given in Equations (10–12).


$$\begin{array}{c} ForQ_{\alpha_{\text{1}}}:\delta_{\text{1}}=Q_{\alpha_{\text{1}}}(s,a)-\tilde{y}\left(r_{safe},s^{\prime },d\right)\\ ForQ_{\alpha_{\text{2}}}:\delta_{\text{2}}=Q_{\alpha_{\text{2}}}(s,a)-\tilde{y}\left(r_{safe},s^{\prime },d\right) \end{array}\}$$ (10)

$$\begin{array}{c} ForQ_{\alpha_{\text{1}}}:{error}_{\text{1}}=\overset{\text{2}}{\underset{\text{1}}{\delta }}={\left(Q_{\alpha_{\text{1}}}(s,a)-\tilde{y}\left(r_{safe},s^{\prime },d\right)\right)}^{\text{2}}\\ ForQ_{\alpha_{\text{2}}}:{error}_{\text{2}}=\overset{\text{2}}{\underset{\text{2}}{\delta }}={\left(Q_{\alpha_{\text{2}}}(s,a)-\tilde{y}\left(r_{safe},s^{\prime },d\right)\right)}^{\text{2}} \end{array}\}$$ (11)

$$\begin{array}{c} ForQ_{\alpha_{\text{1}}}:L\left(\alpha_{\text{1}},B\right)=E_{\left(s,a,r_{safe},s^{\prime },d\right)\sim B}\left[{error}_{\text{1}}\right]\\ ForQ_{\alpha_{\text{2}}}:L\left(\alpha_{\text{2}},B\right)=E_{\left(s,a,r_{safe},s^{\prime },d\right)\sim B}\left[{error}_{\text{2}}\right] \end{array}\}$$ (12)

The term B is the batch drawn from the PER learning, which includes traffic incident data such as vehicle counts, crash severity, and road conditions. For each Q function, the error term is analyzed using the Bellman formulation. The target value incorporates $r_{safe}$ and the min of the next $s_{t}-a_{t}$ pair. The error terms are then squared $\overset{\text{2}}{\underset{\text{1}}{\delta }}$ and $\overset{\text{2}}{\underset{\text{2}}{\delta }}$ to penalize larger differences more heavily, and the loss for each Q-function is averaged. The final $L\left(\alpha_{\text{1}},B\right)$ loss is minimized using gradient descent to make the TD3P-ITC decision, thereby improving the traffic signal control policy and estimating traffic risk through experience-based learning.

#### 3.2.8. Actor network phase.

The actor network update enables the Q-function to converge closer to its true value before the policy is updated, leading to more stable learning. The $\theta_{policy}$ represents the policy network parameters, λ as the learning rate for the policy network, and $J\left(\theta_{policy}\right)$ as the objective function for the policy network, each primary network policy and Q function is paired with a target network in Equation (13).


$$\theta_{policy}\leftarrow \theta_{policy}+\lambda \cdot \nabla_{\theta }J\left(\theta_{policy}\right)$$ (13)

Each primary network is replicated in a target network and updated incrementally, providing stable prediction targets during model training. The actor network takes the $s_{t}$ and outputs the $a_{t}$ representation, which indicates the signal durations. The critic network provides future-state representation for real-time traffic control.

**Fig 5:**
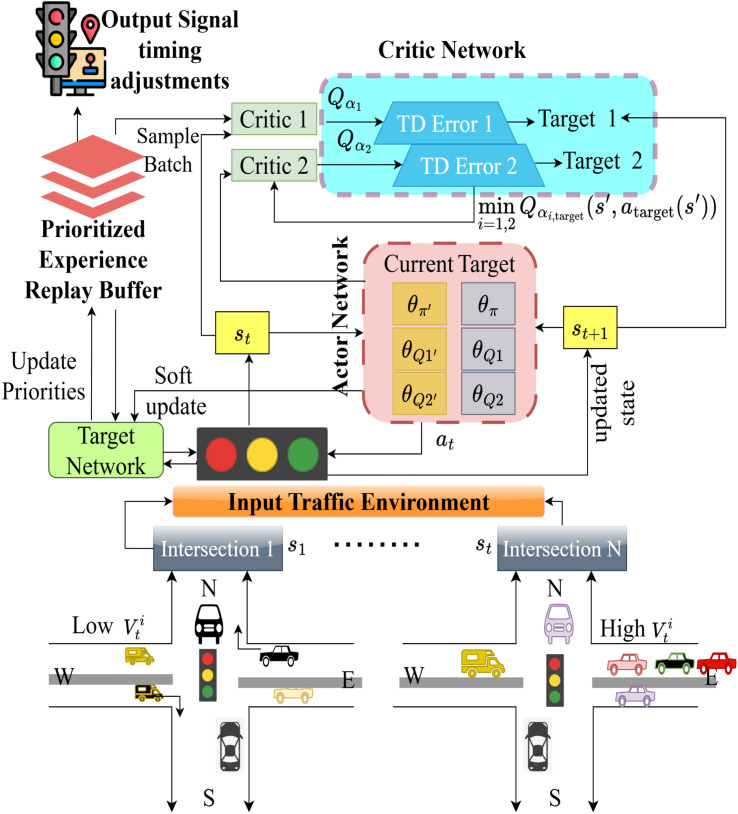
Real-time traffic signal optimization across multiple intersections.

The input traffic environment listed in Fig.5 provides the state $s_{t}$ for multiple intersections with varying traffic densities. The critic network calculates the Q-values $Q_{\alpha_{\text{1}}}$ and $Q_{\alpha_{\text{2}}}$ through two separate estimations, then fed into the TD error to prioritize the experienced replay buffers in high-volume traffic. The actor network generates the optimal signal timing adjustments using current $s_{t}$ and $a_{t}$ pairs in association with target updates $\theta_{{Q\text{1}}^{\prime }}$ and $\theta_{{Q\text{2}}^{\prime }}$ and current policy updates $\theta_{\pi^{\prime }}$. The use of PER enhances learning efficiency by focusing on critical traffic events, and the target network stabilizes updates, allowing for accurate traffic signal optimization in dynamic environments.

The system minimizes waiting times, reduces congestion, and improves throughput across multiple intersections in real-time. The TD3P-ITC training framework is defined below.

**Algorithm: TD3P-ITC Framework Training Procedure**

***Data Required:*** state space $\left[\overset{\text{1}}{\underset{t}{V}},\overset{\text{2}}{\underset{t}{V}},\ldots ,\overset{N}{\underset{t}{V}};\overset{\text{1}}{\underset{t}{Q}},\overset{\text{2}}{\underset{t}{Q}},\ldots .,\overset{N}{\underset{t}{Q}};\overset{\text{1}}{\underset{t}{\Phi }},\overset{\text{2}}{\underset{t}{\Phi }},\ldots ,\overset{N}{\underset{t}{\Phi }};W_{t}\right]$

***Output:*** Optimized policy $\pi_{\theta }$, and Q functions $Q_{\phi \text{1}},Q_{\phi \text{2}}$

Initialize

1. $\pi_{\theta }$, $\pi_{\theta^{\prime }}$

2. $\theta_{Q\text{1}},\theta_{Q\text{2}}$ and targets

3. B=∅, priorities P=∅

4. $\theta^{\prime }\leftarrow \theta ,\phi {\text{1}}^{\prime }\leftarrow \phi \text{1},\phi {\text{2}}^{\prime }\leftarrow \phi \text{2}$

5. ***for*** E = 1 to max_episodes ***do***

6. Init $s_{\text{0}}$ from traffic environment

7. ***for*** t=0toT−1 ***do***

8. $a_{\text{t}}=\pi_{\theta }\left(s_{t}\right)+\epsilon_{t}$

9.  Update $a_{\text{t}}$ in environment

10.  update $-\left(\alpha \cdot \sum_{i=\text{1}}^{N}Q_{i,t+\text{1}}+\beta \cdot \sum_{i=\text{1}}^{N}S_{i,t+\text{1}}+\gamma \cdot {\text{1}}_{accident,t+\text{1}}\right)$

11.  Calculate TD-error using $\delta_{t}$

12.  Store $(s_{t},a_{t},r_{t},s_{t+\text{1}},d_{t})inBwithpriority|\delta_{t}|+\varepsilon$

13.  ***if*** $|B|\geq batch_{size}$***then***

14.   Sample batch with PER probabilities

15.   ***for*** each $\left(s,a,r,s^{\prime },d\right)$ in batch ***do***

16.    calculate$a^{\prime }$

17.    Compute target $y_{t}$

18. ***end for***

19. Update critics: $L_{Q\text{1}}$ *and* $L_{Q\text{2}}$

20. $\phi \text{1}\leftarrow \phi \text{1}-\alpha_{Q}\cdot \nabla \phi \text{1}L_{Q\text{1}}$

21. $\phi \text{2}\leftarrow \phi \text{2}-\alpha_{Q}\cdot \nabla \phi \text{2}L_{Q\text{2}}$

22. iftmodd==0then

23.    Update actor:

$\theta \leftarrow \theta +\alpha_{\pi }\cdot \nabla \theta E[Q_{\phi \text{1}}(s,\pi_{\theta (s)})]$

24.    Soft update targets:

$\theta_{\pi^{\prime }}\leftarrow \tau \cdot \theta_{\pi }+(\text{1}-\tau )\cdot \theta_{\pi^{\prime }}$

$\theta_{{Q\text{1}}^{\prime }}\leftarrow \tau \cdot \theta_{Q\text{1}}+(\text{1}-\tau )\cdot \theta_{{Q\text{1}}^{\prime }}$

$\theta_{{Q\text{2}}^{\prime }}\leftarrow \tau \cdot \theta_{Q\text{2}}+(\text{1}-\tau )\cdot \theta_{{Q\text{2}}^{\prime }}$

25. ***end if***

26. ***end if***

27. ***end for***

28. ***end for***

29. ***return*** $\pi_{\theta },{\text{Q}}_{\phi \text{1}},{\text{Q}}_{\phi \text{2}}$

#### 3.2.9. Prioritization experience replay.

The use of the PER technique ensures that the most critical experiences, which significantly impact traffic flow, are replayed and used to update the learning model. The priority $p_{i}$ of an experience i is usually based on the absolute Temporal Difference error $p_{i}=|\delta |+\varepsilon$ where δ is the TD-error and ε is a small positive constant ensuring all experiences have sampling probability. This PER samples experiences based on priority; higher-priority experiences are those that significantly affect traffic flow, and signal timings are used to update the model. The TD error is calculated as in Equation (14).


$$TD-error=\left(r_{t}+\gamma \cdot min\left(Q_{\alpha_{\text{1}}},Q_{\alpha_{\text{2}}}\right)-Q\left(s_{t},a_{t}\right)\right)$$ (14)

Where $Q_{\alpha_{i}}$ guides the adjustment of the signal timings to minimize delays and congestion while maximizing throughput. The $Q_{\alpha_{\text{1}}}$ and $Q_{\alpha_{\text{2}}}$ estimated by critic one and critic 2, respectively. The sampling probability for each experience is proportional to its priority $p_{i}$ given as $\overset{\alpha }{\underset{t}{P}}$ As a hyperparameter that controls the overestimation bias towards higher-priority experiences, using these prioritized samples enables the model to update its Q-values, ensuring it focuses more on the most critical traffic control experiences. This helps refine the learning process, especially for actions that have a significant impact on traffic flow, such as managing high congestion or adjusting signal timings during heavy traffic.

Priority is determined through proportional prioritization to implement Prioritized Experience Replay (PER). The priority of each transition i is calculated as $p_{i}=(|\delta_{i}|+\varepsilon {)}^{\alpha_{\text{PER}}}$where $\delta_{i}$ is the temporal-difference error of the critic networks, ε=10–6, prevents zero-priority, and $\alpha_{\text{PER}}$=0.6 is the parameter used to interact with the degree of prioritization. The priorities are updated immediately after each learning update, using new calculated TD errors. The transitions are sampled based on $P(i)=\frac{p_{i}}{\sum_{j}\;\;p_{j}}$ to avoid the bias caused by the uneven sampling, the sample loss of the critic is corrected with importance-sampling weights $w_{i}={\left(\frac{\text{1}}{N\cdot P(i)}\right)}^{\beta_{\text{PER}}}$, where N is the size of a replay buffer. Practically, it is the exponent of the correction term $\beta_{\text{PER}}$ that is linearly annealed between 0.4 and 1.0 during training, and the weights are normalized by $w_{i}$ to stabilize the optimization, which allows a reasonable balance between high-error transition exploration and an unbiased value estimate.

**Fig 6:**
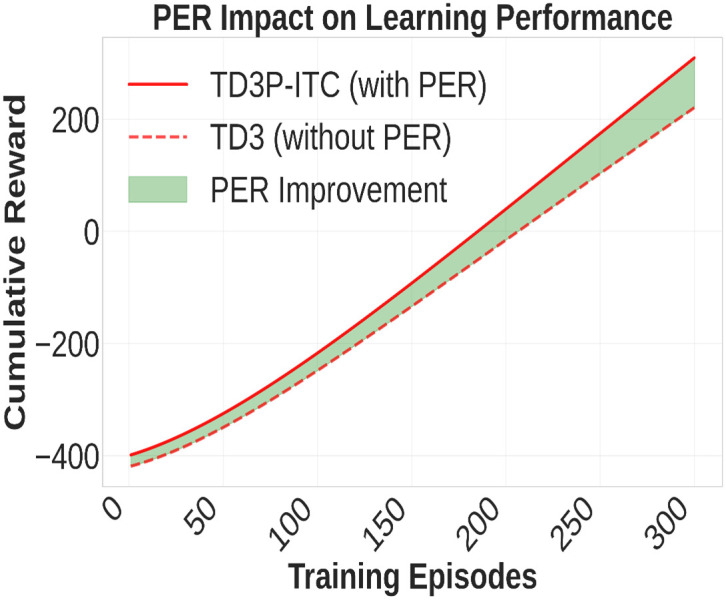
Impact of PER on learning performance.

In Fig 6, PER accelerates learning by prioritizing critical traffic experiences with high temporal-difference errors, ensuring the system focuses on high-impact scenarios, such as emergency vehicle passages or congested areas, through probability-weighted sampling. This notable improvement in learning efficacy is achieved when employing PER within the TD3P-ITC framework. The TD3P-ITC with PER consistently outperforms the TD3 model without PER, as evidenced by the higher cumulative reward increase across training episodes. The shaded green region in Fig 6 illustrates the improvement achieved with PER, demonstrating its effectiveness in prioritizing experiences for enhanced learning. This study demonstrates the efficacy of PER-based reinforcement learning for controlling urban traffic signals. The cumulative reward for TD3P-ITC rises more quickly to higher levels, which means that policy optimization is more effective during training. Its practical significance lies in its ability to improve real-time traffic flow while ensuring safety, making it a strong candidate for future ITS applications.

In summary, the unique TD3P-ITC for real-time traffic signal optimization across multi-intersection networks provides an effective control of real-time traffic signal decisions. For stable learning in dynamic traffic situations, the system uses PER, which prioritizes essential traffic scenarios with high temporal-difference errors to efficiently use experience. The system employs dual normalization algorithms to handle a 12-feature state space, incorporating normalized traffic volume, inferred queue lengths, signal phases, and environmental conditions for context-aware decision-making across various traffic patterns. By balancing queue minimization, stop reduction, and accident prevention with weighted penalty terms (α, β, γ), a multi-objective reward function optimizes signal timing to achieve both safety and performance. Dynamic action-space controllers adjust the green signal phase based on queue thresholds and throughput measurements to regulate traffic flow within the modular architecture.

## 4. Results and discussion

The proposed TD3P-ITC research is conducted in a high-performance computing environment with multicore processors and GPUs for accelerated computation. Software tools include Python, for data processing, along with TensorFlow and PyTorch. The simulation is performed using the SUMO traffic simulation platform for real-time modeling. The system is equipped with 64GB of RAM and Nvidia RTX GPUs for efficient model training and evaluation. During the training period, the TD3P-ITC model’s GPU memory never exceeded 6 GB, and the test run converged to within 5,000 episodes in around 3.2 hours. To ensure reproducibility, all tests were conducted using fixed random seeds for the Python runtime, NumPy, and deep learning resources. The results provided should reflect the average performance after running many trials with different random seeds. Using realistic traffic demand distributions, vehicle composition, signal statuses, and environmental conditions, the Kaggle dataset is used to initialize and parameterize the traffic simulation environment. A SUMO interaction microscopic simulator employs a reinforcement learning agent. In SUMO, the agent is not taught using a static dataset; rather, it learns state transitions, rewards, and experiences through online interactions with its environment.

The SUMO microscopic simulator implemented a traffic simulation environment for a multi-intersection metropolitan network, including residential, business, highway, mixed-use, and transport-hub junctions. Time-varying flow profiles generated traffic demand and simulated peak and off-peak situations, while non-homogeneous Poisson processes predicted vehicle arrival. Because publicly available data does not provide actual origin-destination (OD) information, route choice was determined using predetermined probabilistic route distributions, which maintain realistic route diversity while controlling demand. Based on observed data, the car composition was sampled. Changes in vehicle dynamics parameters modeled weather influences showed that rain, fog, and wind increased speed decrease and acceleration noise, indirectly increasing queue building and accident probability. For a fair comparison, demand patterns, routing logic, and weather modifiers were maintained between the baseline and proposed techniques.

Table 3 lists the primary parameters and ranges or default settings of the TD3P-ITC traffic signal control model. Q-Network and Policy Network parameters determine learning rate ($\eta_{\text{Q}}$, $\alpha_{\pi }$) and update frequency across both networks. During training, exploration parameters like ∈ and σguide the agent’s exploration. The PER parameters α,β, and $\delta_{min}$ regulate sampling. Penalties for queue, halt, and safety change reward function components. The target network update rule and Q-Loss function guide the model’s learning and convergence criteria, ensuring the agent’s persistent improvement.

**Table 3:** Hyperparameter tuning settings for TD3P-ITC framework.

Component	Parameter	Range/Value	Default
**Q-Networks**	$\alpha_{\text{Q}}$	$\left[{\text{10}}^{-\text{5}},{\text{10}}^{-\text{3}}\right]$	0.001
	τ	$\left[{\text{10}}^{-\text{4}},{\text{10}}^{-\text{2}}\right]$	0.005
**Policy Network**	$\alpha_{\pi }$	$\left[{\text{10}}^{-\text{5}},{\text{10}}^{-\text{3}}\right]$	0.0003
	Update Frequency	Every 22 critic updates	Every two updates
**Exploration**	∈	N(0,0.1)	0.1
	σ	[0.1,0.3]	0.2
	c	[0.1,1.0]	0.5
**PER**	$\alpha_{PER}$	[0.4,0.8]	0.6
	β	[0.4,1.0]	0.6
	$\delta_{min}$	[0.005,0.1]	0.01
	B	[32,128]	64
	Maximum size	20000	20000
**Discount Factor**	γ	[0.9,0.99]	0.99
**Reward Function Components**	Queue Function	[0.2,0.6]	0.4
	Stop Function	[0.2,0.6]	0.3
	Safety Penalty Weight	[−15.0, −5.0]	−10.0
	δ	[0.2,0.6]	0.3
**Target Network Updates**	Target Networks Update Rule	$\theta_{\pi^{\prime }}\leftarrow \tau \cdot \theta_{\pi }+(\text{1}-\tau )\cdot \theta_{\pi^{\prime }}$	τ=0.005
**Q-Loss Calculation**	Q-Loss Function	L	–
	Target Value Calculation	$y_{t}$	–
**General Updates**	$\theta_{policy}$	Every 22 critic updates	Every two updates
	Convergence Criteria	<5000episodes	

The 2,000 observations are the size of the time-indexed traffic record set used to parameterize and establish the traffic simulation environment, not the reinforcement learning agent’s cumulative learning samples. In an interactive simulation environment, each episode is broken down into several sequential decisions, where the agent chooses when to take a signal and observes state transitions and rewards. This means that one episode yields many state-action-reward-next-state tuples, and the total number of experiences saved can be huge relative to the original dataset.

The replay buffer of 20,000 is the maximum number of interaction-induced transitions stored to learn off-policy, regardless of raw dataset size. Instead of storing 2,000 dataset observations, the replay buffer contained interaction-based transitions. The AI was trained in a simulated environment on traffic conditions using a single observation and executed signal control actions over multiple time steps per episode. Each action caused a state-action-reward-next-state transition, which was added to the replay buffer with each episode. The buffer capacity of 20,000 measures interaction-based experiences with multiple simulation rollouts, not the baseline dataset’s records. The convergence of fewer than 5,000 episodes suggests that the learnt policy utilized identical data samples during successive rollouts under varying traffic conditions and control actions. In classic off-policy actor-critic reinforcement learning, the richness and variety of interaction-based events determine learning progress over dataset cardinality.

The fixed dataset is used to bootstrap and parameterize a microscopic traffic simulation, not for offline learning. Through signal timing actions, the agent dynamically affects vehicle movement, queue evolution, and delay, producing new state transitions and rewards at each decision step. As with reinforcement learning, learning is interaction-induced, based on experiences accumulated throughout simulation rollouts, and is no longer limited by the quantity of raw dataset observations.

A simulator was constructed, and its parameters were set using the data, without using offline reinforcement learning. The dataset includes traffic demand profiles, statistics on vehicle composition, information on signal states, and ambient conditions that can be used to set up and run a microscopic simulation. In contrast to policy learning on fixed trajectories, this simulator enables typical interaction-based reinforcement learning by allowing the agent to interact online through control actions to generate new state transitions and rewards.

### 4.1. Traffic control simulation

**Fig 7:**
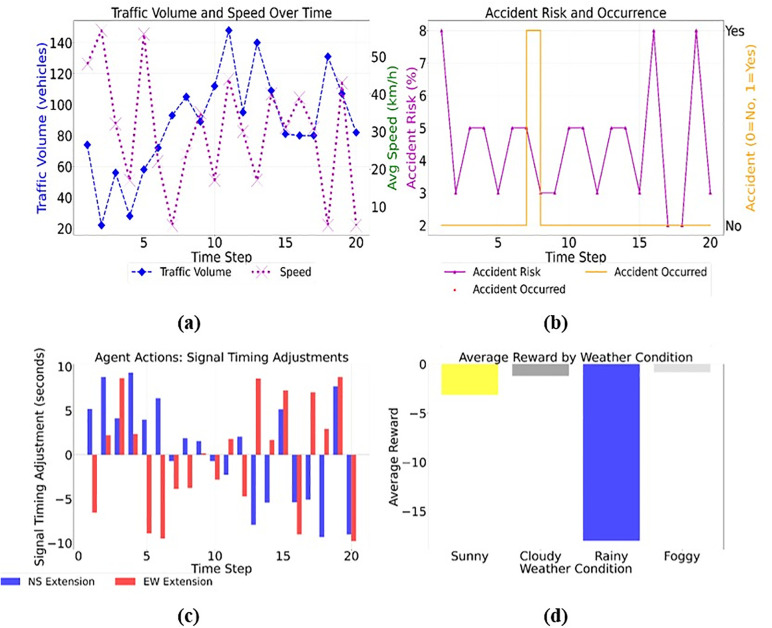
Simulation Analysis of Traffic Control and Agent Actions Over Time a) Traffic Volume and Speed, b) Accident Risk and Occurrence, c) Signal Timing Adjustments, and d) Average Reward by Weather Condition.

The model utilizes PER and TD3 to focus on high-impact traffic situations, enabling faster learning (Fig 7a-Fig 7d).

The traffic volume and average vehicle speed over time, with distinct traffic behaviors varying across signal phases. The accident risk and recurrence highlight moments when accidents occur due to environmental factors. The third plot illustrates decision-making for timing segments by showing the NS and EW signal phase extensions. The effect of weather conditions on reward values demonstrates how different weather patterns affect traffic control. TD3P-ITC is likely to work more effectively than other traffic control systems, such as SMART and M2SAC, as it learns various traffic conditions, accidents, and weather effects. It provides accurate traffic data, including the number of cars on the road, their speed, and the frequency of accidents. It also takes into account how weather conditions affect traffic. The agent changes the length of traffic lights based on these variables and random actions.

### 4.2. Comparative performance analysis

The selection of existing models SMART [18], FPPO [19], D3QN [22], and M2SAC [26] for comparison with the proposed TD3P-ITC model is strategically chosen to showcase its efficiency in real-time traffic signal control. The SMART [18] model is effective at reducing mean travel time; however, it incurs high computational costs and scalability issues when applied to large urban networks. TD3P-ITC addresses these issues through PER and clipped double Q-learning. FPPO [19], known for its stability during policy updates, lacks robustness to handle rapidly changing traffic conditions. This limitation is overcome by TD3P-ITC, which uses delayed updates and target policy smoothing to maintain stability while adapting to dynamic environments. D3QN [22] balances efficiency and safety, but it is prone to Q-value overestimation bias, leading to slightly longer waiting times. M2SAC [26] faces limitations in complex traffic environments due to its multi-agent approach. In contrast, the proposed TD3P-ITC enhances traffic flow stability, scalability, and efficiency, necessitating adaptive, real-time traffic signal control to mitigate congestion and reduce waiting times.

All baseline methods—SMART, FPPO, D3QN, and M2SAC—were reimplemented in the same simulation environment to ensure a controlled, fair comparison. The hyperparameter settings in both models were derived from the original methodological settings described in the reference papers. These settings included schedules for learning rate, discounting, target network update, exploration or entropy regularization, and replay buffer, among others. The models’ control structures were kept unchanged from their original formulations. For example, FPPO used centralized critics, D3QN used duelling architectures, M2SAC used entropy-regularized actor-critic updates, and SMART used rule-based actuation logic. To ensure accurate performance results, only parameters affected by the environment were considered, such as the state dimensionality and action limitations. No further hyperparameter modification was employed.

Each baseline approach (SMART, FPPO, D3QN, and M2SAC) was evaluated under identical simulation settings to ensure a fair, controlled comparison. In particular, each model had the same SUMO network topology, traffic demand model, vehicle makeup, routing logic, weather, and signal safety constraints. Traffic generation, route assignment, and the simulator’s stochasticity used the same random seed. Minor implementation adjustments were made to align the state dimensionality, action boundaries, and decision intervals with the standard experimental setup. No algorithm-specific performance tuning or incentive reparameterization was done. The uniform evaluation approach would ensure that learning and control strategies, not environmental and implementation biases, explain performance differences.

#### 4.2.1. Average waiting time.

**Fig 8:**
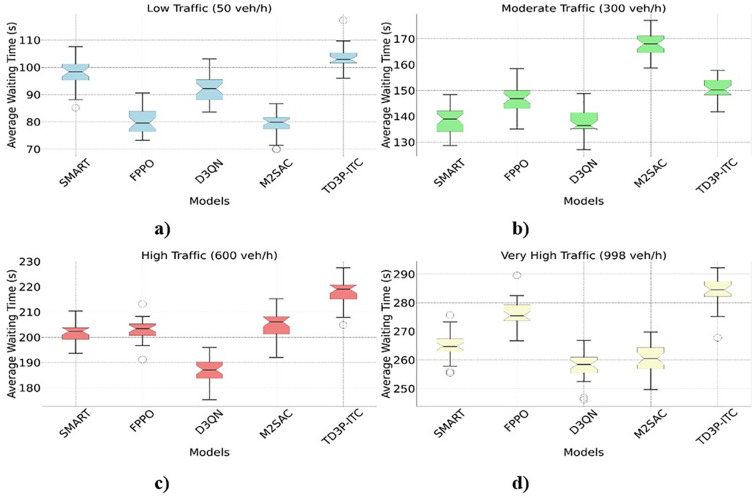
Average Waiting Time for Different Traffic Signal Control Models Across Various Traffic Scenarios a) Low Traffic (50 Veh/h), b) Moderate (300 Veh/h), c) High Traffic (600 Veh/h), and d) Very High Traffic (998 veh/h).

The plot shown in Fig 8a-Fig 8d displays the variation in waiting times for each model under different traffic volumes, with the box showing the interquartile range and the median line indicating the central tendency. The markers represent the range of waiting times, and the outliers are indicated. The adjusted waiting times suggest that the combination of traffic flow dynamics and model performance is crucial for optimizing real-time traffic signals. The average waiting time for five different traffic signal control models (SMART, FPPO, D3QN, M2SAC, and TD3P-ITC) under four different traffic conditions: Low Traffic (50 veh/h), Moderate Traffic (300 veh/h), High Traffic (600 veh/h), and Very High Traffic (998 veh/h). The box color indicates the traffic state, and each boxplot shows the average wait time for a specific situation. The data is simulated with noise to make it more realistic, and the traffic-volume scaling factors ensure that waiting times increase as traffic density rises. The graphs show the median, IQR, and outliers, making it easy to compare how well each model did in different situations. This picture illustrates how the TD3P-ITC model compares to other models in terms of reducing wait times, particularly when traffic conditions change.

#### 4.2.2. Throughput efficiency analysis.

The TD3P-ITC model achieves superior performance across various weather conditions, intersection types, and vehicle types, as shown in Table 4, due to its novel integration of TD3 with PER, which adapts traffic flow and safety. The 4.0% improvement over existing models M2SAC, D3QN, FPPO, and SMART is calculated based on throughput enhancement. Variant 1: Weather Conditions shows that TD3P-ITC consistently outperforms other models, with a 4.0% improvement in sunny weather and better handling of rain and fog. Variant 2: Intersection Types shows that TD3P-ITC performs best on highways and at transit hubs, where it can handle significantly more traffic. Finally, Variant 3: Vehicle sorts shows that TD3P-ITC performs well across cars, trucks, and buses, and outperforms the other versions across all vehicle types. The TD3P-ITC model adapts more effectively and performs better across varied traffic conditions, as evidenced by higher throughput and improved management of environmental and traffic complexity. By employing stability techniques, the framework reduces the likelihood of overestimation. These processes enable TD3P-ITC to make more informed and stable decisions about traffic signals, particularly when traffic is complex. The PER enhances learning efficiency by prioritizing experiences with a significant impact, such as heavy traffic or situations where accidents are more likely. This helps people learn more quickly and adapt more effectively to various traffic conditions.

**Table 4:** Throughput comparison of traffic signal control methods.

Variant 1: Weather Conditions								
Method	Sunny		Rainy	Foggy		Cloudy		Windy
**TD3P-ITC**	4,089 ± 67		3,234 ± 98	2,897 ± 112		3,678 ± 78		3,456 ± 89
**SMART [18]**	3,612 ± 89		2,745 ± 143	2,398 ± 167		3,234 ± 123		2,987 ± 134
**FPPO [19]**	3,789 ± 78		2,934 ± 124	2,567 ± 145		3,412 ± 98		3,156 ± 112
**D3QN [22]**	3,734 ± 92		2,856 ± 138	2,489 ± 159		3,367 ± 114		3,089 ± 127
**M2SAC [26]**	3,923 ± 81		3,089 ± 118	2,734 ± 132		3,567 ± 89		3,289 ± 103
**Variant 2: Intersection Types**								
**Method**	**Residential**		**Commercial**	**Highway**		**Mixed-Use**		**Transport Hub**
**TD3P-ITC**	2,834 ± 78		4,267 ± 123	5,189 ± 167		3,678 ± 94		4,523 ± 134
**SMART [18]**	2,456 ± 98		3,689 ± 156	4,234 ± 198		3,167 ± 134		3,834 ± 178
**FPPO [19]**	2,634 ± 87		3,923 ± 143	4,567 ± 189		3,389 ± 118		4,089 ± 167
**D3QN [22]**	2,567 ± 92		3,834 ± 149	4,456 ± 192		3,234 ± 127		3,967 ± 174
**M2SAC [26]**	2,723 ± 82		4,089 ± 132	4,834 ± 178		3,523 ± 103		4,267 ± 156
**Variant 3: Vehicle Types**								
**Method**		**Cars**			**Trucks**		**Buses**	
**TD3P-ITC**		3,456 ± 89			2,234 ± 123		1,789 ± 98	
**SMART [18]**		3,089 ± 112			1,867 ± 167		1,456 ± 134	
**FPPO [19]**		3,234 ± 98			1,978 ± 149		1,589 ± 118	
**D3QN [22]**		3,167 ± 105			1,923 ± 156		1,534 ± 127	
**M2SAC [26]**		3,334 ± 92			2,089 ± 138		1,678 ± 112	

#### 4.2.3. Queue length minimization.

**Fig 9:**
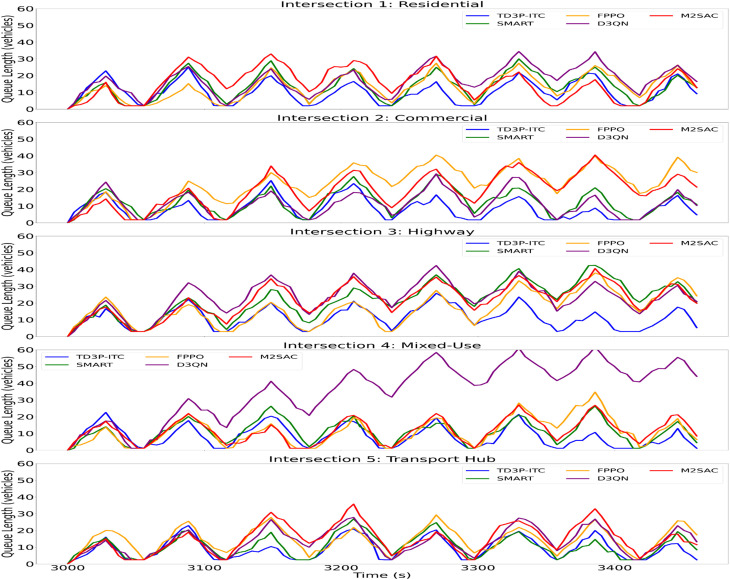
Queue Length Analysis on Various Traffic Intersections: a) Residential, b) Commercial, c) Highway, d) Mixed-Use, and e) Transport Hub.

The illustrated Fig 9a-Fig 9e shows the lengths of car queues at five distinct types of intersections: Residential, Commercial, Highway, Mixed-Use, and Transport Hub. It utilizes multiple traffic signal control algorithms, including TD3P-ITC, SMART, FPPO, D3QN, and M2SAC.

The results show that TD3P-ITC outperforms other models at reducing queues, especially in high-traffic areas such as highways and transport hubs. The Q-value update rule ensures the model continuously refines its decision-making, resulting in more efficient traffic flow. The changes in queue lengths over time demonstrate how each algorithm responds to varying traffic conditions. TD3P-ITC keeps queues that are substantially shorter and more stable. These results align with the objectives of real-time traffic signal control research, which seeks to enhance traffic flow and mitigate congestion. From this analysis, a 22% reduction in peak queue lengths at the Transport Hub intersection and a 25% reduction at the highway intersection are demonstrated, while managing high-traffic environments by optimizing traffic signal control and minimizing congestion.

#### 4.2.4. Training model convergence analysis.

**Fig 10:**
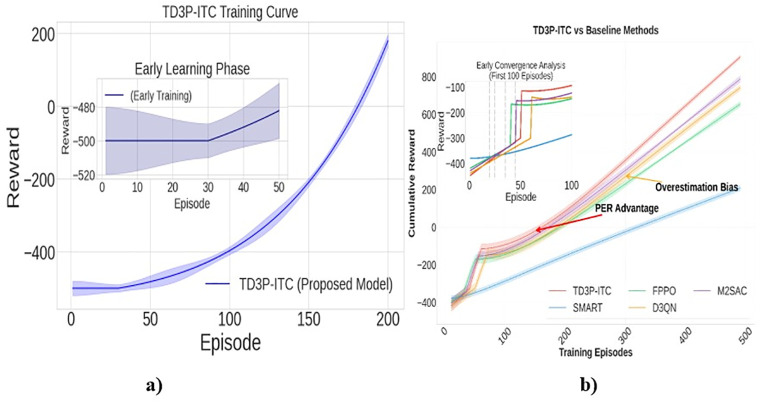
Training Performance Analysis: a) TD3P-ITC Model and b) Convergence.

During training, the agent $a_{t}$ receives rewards based on how effectively it reduces congestion, optimizes traffic flow, and minimizes vehicle waiting time. After several iterations, called training episodes, the TD3P-ITC model converges to the optimal signal timings expressed as $\overset{*}{\underset{L_{i}}{S}}=\text{arg}\underset{S_{L_{i}}}{\text{max}}\sum_{t}r_{t}$ for lane $L_{i}$. The model selects optimal timing settings that maximize throughput, minimize queue lengths, and reduce average waiting times in intersection scenarios, thereby effectively enhancing real-time traffic flow. During the inference shown in Fig. 10a and Fig. 10b, the trained policy network provides real-time traffic signal control decisions for each intersection, dynamically optimizing signal timing based on observed traffic conditions.

#### 4.2.5. Analysis with baseline models.

Table 4 presents the evaluation of traffic signal control algorithms, examining the impact of SMART [18], FPPO [19], D3QN [22], and M2SAC [26] on key evaluation metrics, including wait time, throughput, queue length, and accident rate. The differences between the traffic signal control algorithms. p < 0.001**. These markers help determine how reliable performance increases are by examining factors such as wait time, throughput, and accident rate. In traffic signal control, lower p-values (e.g., p < 0.001*) indicate a higher likelihood that the algorithm is responsible for the observed performance benefits (e.g., shorter wait times, reduced congestion) rather than random chance. This helps ensure that the algorithm operates effectively and reliably in real-life traffic management.

The traffic simulator reduced simulated accident incidences by 17.9% compared to baseline controllers under the same traffic demand and environmental conditions. Accident counts are recorded when a simulator’s safety model flags a collision or close contact. This measure is based on the empirical number of accident incidents per episode averaged over evaluation runs, not cumulative reward or punishment magnitude. Table 5 was statistically tested using two-tailed independent t-tests on the average per-episode metrics, with n = 50 evaluation episodes per method. The degrees of freedom for the waiting time, throughput, queue length, and accident rate were df = 98 in all paired comparisons. The related test values ranged from t = 3.12 to t = 6.47, and the effect size (Cohen d) ranged from 0.62 to 1.28, indicating medium to large practical significance. A Bonferroni correction was applied to a series of multiple pairwise comparisons of the four baselines and 4 performance metrics (K = 16), yielding an adjusted significance level of 0.0031.

**Table 5:** Statistical significance analysis (Two-tailed t-test Results).

Metric	SMART	FPPO	D3QN	M2SAC
**Wait Time**	p < 0.001***	p < 0.001***	p < 0.001***	p < 0.001***
**Throughput**	p < 0.001***	p < 0.001***	p < 0.001***	p = 0.002**
**Queue Length**	p < 0.001***	p < 0.001***	p < 0.001***	p < 0.001***
**Accident Rate**	p < 0.001***	p < 0.001***	p < 0.001***	p = 0.018*

#### 4.2.6. Complexity analysis.

**Fig 11:**
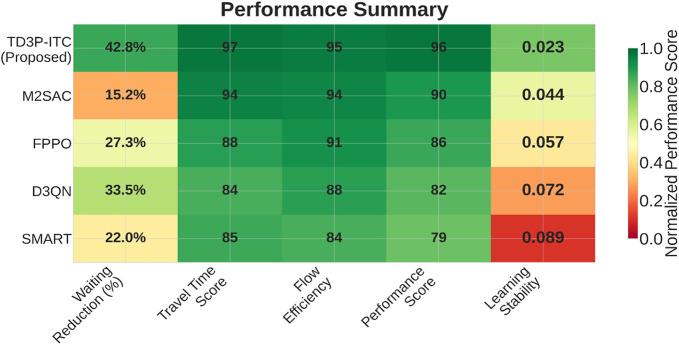
Performance summary of research models.

The computational complexity analysis provides that the TD3P-ITC system achieves better performance with space complexity $O\left(l\times H^{\text{2}}+M\times S\right)$ and inference time complexity of $O\left(l\times H^{\text{2}}+H\times (S+A)\right)$, enabling real-time operation with faster decision-making. The state dimension S indicates the number of features per intersection with queue length, vehicle counts, waiting times, and current signal phases and duration. The action dimension A suggests the number of control signal parameters for phase timing adjustments. With five intersection points, 20 state features, and 256 hidden neurons H with layers l, the memory buffer remains manageable. Training complexity scales per B as batch size for training termed as $O\left(B\times \left(l\times H^{\text{2}}+H\times (S+A)\right)\right)$ during each training time step with PER sampling efficiency of O(logM) with memories M range as ${\text{1}\text{0}}^{\text{5}}-{\text{1}\text{0}}^{\text{6}}$ experiences. Communication overhead ranges from the number of intersections N in the traffic network as O(N×S) for coordination to $O\left(N^{\text{2}}\times S\right)$ for centralized approaches. The heatmap in Fig 11 shows the performance of five traffic signal control models across key metrics, including waiting reduction, travel time score, flow efficiency, performance score, and learning stability. TD3P-ITC achieves the best waiting reduction (42.8%) as a cumulative performance gain across benchmark and travel time scores (97), but it also exhibits the lowest learning stability (0.023). On the other hand, M2SAC reduces waiting time by 15.2%. However, it is 27.6% behind TD3P-ITC in terms of waiting reduction and 3% behind in travel time score, utilizing the clipped double Q learning stability technique. In terms of flow efficiency, the existing FPPO and D3QN perform better in training, while TD3P-ITC does a better job at reducing waiting time and improving performance scores. The traditional method, SMART, has the worst results, with a 22.0% decrease in waiting time and lower scores in other areas. TD3P-ITC outperforms SMART in terms of waiting reduction (20.8%) and travel time Score (13.5%), indicating its effectiveness in optimizing traffic signals in real time.

In addition to the asymptotic complexity study, an empirical timing analysis was conducted to assess real-time viability and to infer recent deployment. The taught TD3P-ITC policy decides at each intersection in 3–5 ms on conventional GPU processors and in less than 20 ms on a CPU-only edge traffic controller model, which is suitable for real-time operation with a normal signal update rate (>1s). The offline training takes 3.2 hours to converge over 5,000 episodes; however, the online operation remains unaffected. The computational delay of inference scales linearly with the intersection number; therefore, lightweight actor networks can execute inference in real time with fixed dimensionality. The proposed system can be deployed on standard traffic management equipment, with central or offline learning and low-latency inference at runtime.

The continuous-control Markov Decision Process for traffic signal control reduces vehicle waiting time and queue length, and ensures traffic safety under dynamic, uncertain traffic conditions. Actor-critic algorithms are excellent for this challenge because the model directly optimizes continuous green-phase timings, unlike discrete-action models, which pick a time phase from a timing schedule. The bias toward overestimation in the continuous action space, introduced by clipping and updating, and the delayed policy updates in double Q-learning influence the use of TD3. The instability of value estimates causes oscillatory signal behaviour and cascading intersection congestion, making such qualities useful in traffic signal regulation. It is used with PER to balance traffic with high-impact, low-frequency events, such as congestion peaks and high-risk accidents. Sample transitions based on temporal-difference error to highlight states that have the greatest impact on network-level performance, thereby improving sample efficiency and convergence stability.

The state representation covers instantaneous traffic conditions and contextual elements that affect signal operation. Congestion is measured by inferred queue length and normalized traffic volume, while signal-phase encoding ensures policy-awareness during the learning phase. Weather affects vehicle speed and accident risk. Normalising features ensures numerical stability during training and prevents value-function approximation bias towards large inputs. To balance efficiency and safety, the rewarding function is a weighted multi-objective signal. Stop-related penalties and wait length reduce congestion and improve traffic flow, and accidents will deter hazardous signal layouts. This formulation avoids hard-coded traffic algorithms and allows the agent to learn adaptive trade-offs as it operates in the system. To guarantee scale consistency across rewards and safety breach penalties compared to efficiency advantages, weighting coefficients are established. The suggested controller is tested in a tiny simulated traffic environment with varied traffic, weather, and vehicle compositions over a multi-intersection network. In the same conditions, the simulated environment enables controlled analysis of policy behaviour and benchmarking against current methodologies. This research examines learning dynamics and control stability at a small scale across a few crossings; however, it lacks structural limits that would prevent its application to larger networks. The methodology defines a clear relationship among control goals, the learning architecture, and the evaluation protocol, enabling performance gains to be attributed to the proposed TD3P-ITC design rather than to implicit assumptions or heuristic adjustments.

## 5. Conclusion

The presented TD3P-ITC framework represents a significant step towards fully autonomous ITS that can adapt to dynamic conditions while ensuring optimal safety and efficiency outcomes. The fusion of TD3’s stability mechanisms with PER’s learning policy creates a robust framework that is optimized for safety-critical traffic applications. The multidimensional aspect of the TD3P-ITC module addresses specific aspects of traffic control, from state processing to ensuring data quality and dynamic action controllers managing real-time signal timing adjustments. The TD3P-ITC framework significantly advances intelligent traffic control by employing DRL for dynamic signal optimization. The results demonstrate that a 4% improvement in throughput efficiency and a 9.6% reduction in wait time are achieved by efficiently learning optimal traffic signal timings through state and dynamic action space representation and a reward function. The Q-value stability and delayed policy updates help avoid overestimation of signal benefits, ensuring smoother traffic signal control. This results in 14% faster convergence and 7.2% higher sample efficiency, validating its practical implications for real-world traffic systems. At high-demand crossings, experimental results reveal a considerable reduction in wait length (up to 25%) and a drop in experimentally-emulated accident rates of 17.9%. Limited data samples may not capture long-term traffic patterns that impact seasonal analysis, potentially leading to overfitting to specific periods and a limited variety in accident types and severity analysis. The framework’s modular design offers flexibility for future expansion, including large-scale urban validation and multi-modal integration.

## References

[1] Tan X, Zhou Y, Jiao X. Traffic signal control based on deep reinforcement learning using state fusion and trend reward. Engineering Applications of Artificial Intelligence. 2025;159:111701.

[2] Wang L, Zhang G, Yang Q, Han T. An adaptive traffic signal control scheme with Proximal Policy Optimization based on deep reinforcement learning for a single intersection. Engineering Applications of Artificial Intelligence. 2025;149:110440.

[3] Bálint K, Tamás T, Tamás B. Deep reinforcement learning based approach for traffic signal control. Transportation Research Procedia. 2022;62:278–85.

[4] Huang Z. Reinforcement learning based adaptive control method for traffic lights in intelligent transportation. Alexandria Engineering Journal, 2024;106:381–91.

[5] Haydari A, Yılmaz Y. Deep reinforcement learning for intelligent transportation systems: A survey. IEEE Transactions on Intelligent Transportation Systems, 2020;23(1):11–32.

[6] Li Z, Yu H, Zhang G, Dong S, Xu CZ. Network-wide traffic signal control optimization using a multi-agent deep reinforcement learning. Transportation Research Part C: Emerging Technologies. 2021;125:103059.

[7] Xu D, Liao X, Yu Z, Gu T, Guo H. Robustness enhancement of deep reinforcement learning-based traffic signal control model via structure compression. Knowledge-Based Systems. 2025;310:113022.

[8] Pang A, Wang M, Chen Y, Pun MO, Lepech M. Scalable reinforcement learning framework for traffic signal control under communication delays. IEEE Open Journal of Vehicular Technology. 2024;5:330–43.

[9] Xu T, Pang Y, Zhu Y, Ji W, Jiang R. Real-time driving style integration in deep reinforcement learning for traffic signal control. IEEE Transactions on Intelligent Transportation Systems. 2025;26:11879–92.

[10] Yang T, Fan W. Transit signal priority under connected vehicle environment: Deep reinforcement learning approach. Journal of Intelligent Transportation Systems. 2025;29(5), 505–17.

[11] Liu Y, Liang J, Zhang Y, Gong P, Luo G, Yuan Q, Li J. GlobalLight: Exploring global influence in multi-agent deep reinforcement learning for large-scale traffic signal control. Neurocomputing. 2025;637:130065.

[12] Swapno SMMR, Nobel SMN, Meena P, Meena VP, Azar AT, Haider Z, et al. A reinforcement learning approach for reducing traffic congestion using deep Q learning. Sci Rep. 2024;14(1):30452. doi: 10.1038/s41598-024-75638-0 39668197 PMC11638258

[13] Kolat M, Kővári B, Bécsi T, Aradi S. Multi-agent reinforcement learning for traffic signal control: A cooperative approach. Sustainability. 2023;15(4):3479.

[14] Yang T, Fan W. Enhancing robustness of deep reinforcement learning based adaptive traffic signal controllers in mixed traffic environments through data fusion and multi-discrete actions. IEEE Transactions on Intelligent Transportation Systems. 2024;25(10):14196–208.

[15] Bouktif S, Cheniki A, Ouni A, El-Sayed H. Deep reinforcement learning for traffic signal control with consistent state and reward design approach. Knowledge-Based Systems. 2023;267:110440.

[16] Zhang M, Wang D, Cai Z, Huang Y, Yu H, Qin H, Zeng J. EGLight: enhancing deep reinforcement learning with expert guidance for traffic signal control. Transportmetrica A: Transport Science. 2025:1–27.

[17] Wu Q, Wu J, Shen J, Du B, Telikani A, Fahmideh M, Liang C. Distributed agent-based deep reinforcement learning for large scale traffic signal control. Knowledge-based systems. 2022;241:108304.

[18] Fereidooni Z, Palesi LAI, Nesi P. Multi-agent optimizing traffic light signals using deep reinforcement learning. IEEE Access. 2025;13:106974–106988.

[19] Li M, Pan X, Liu C, Li Z. Federated deep reinforcement learning-based urban traffic signal optimal control. Sci Rep. 2025;15(1):11724. doi: 10.1038/s41598-025-91966-1 40188158 PMC11972306

[20] Wang T, Zhu Z, Zhang J, Tian J, Zhang W. A large-scale traffic signal control algorithm based on multi-layer graph deep reinforcement learning. Transportation Research Part C: Emerging Technologies. 2024;162:104582.

[21] Bouktif S, Cheniki A, Ouni A, El-Sayed H. Parameterized-action based deep reinforcement learning for intelligent traffic signal control. Engineering Applications of Artificial Intelligence. 2025;159:111422.

[22] Zhang G, Chang F, Jin J, Yang F, Huang H. Multi-objective deep reinforcement learning approach for adaptive traffic signal control system with concurrent optimization of safety, efficiency, and decarbonization at intersections. Accid Anal Prev. 2024;199:107451. doi: 10.1016/j.aap.2023.107451 38367397

[23] Cao K, Wang L, Zhang S, Duan L, Jiang G, Sfarra S, Jung H. Optimization control of adaptive traffic signal with deep reinforcement learning. Electronics. 2024;13(1):198.

[24] Cai C, Wei M. Adaptive urban traffic signal control based on enhanced deep reinforcement learning. Sci Rep. 2024;14(1):14116. doi: 10.1038/s41598-024-64885-w 38898047 PMC11186829

[25] Bao J, Wu C, Lin Y, Zhong L, Chen X, Yin R. A scalable approach to optimize traffic signal control with federated reinforcement learning. Sci Rep. 2023;13(1):19184. doi: 10.1038/s41598-023-46074-3 37932347 PMC10628245

[26] Zhang X, Chan LS, Nassir N, Sarvi M. Towards fair lights: A multi-agent masked deep reinforcement learning for efficient corridor-level traffic signal control. Communications in Transportation Research. 2025;5:100203.

[27] Yazdani M, Sarvi M, Bagloee SA, Nassir N, Price J, Parineh H. Intelligent vehicle pedestrian light (IVPL): A deep reinforcement learning approach for traffic signal control. Transportation research part C: emerging technologies. 2023;149:103991.

[28] Zhao J, Yao T, Zhang C, Shafique MA. Signal control for overflow prevention at intersections using partial connected vehicle data. Transportmetrica A: Transport Science. 2024:1–31.

[29] Shi Y, Guler SI, Zhao J, Zhu J, Yang X. Increasing signalized intersection capacity with flexible lane design. Transportation Research Part C: Emerging Technologies, 2025;173:105054.

[30] Jalil K, Xia Y, Zhao J. Advancements in collision avoidance techniques for internet-connected vehicles: A comprehensive review of methods and challenges. Engineering Applications of Artificial Intelligence, 2025;160:111836.

[31] Dataset. https://www.kaggle.com/datasets/smmmmmmmmmmmm/smart-traffic-management-dataset

